# Microfibers Accumulation within a Mediterranean Submesoscale Cyclone

**DOI:** 10.1021/acs.est.5c13987

**Published:** 2026-01-14

**Authors:** Giovanni Testa, Giuseppe Suaria, Andrea Paluselli, Salomé La Ragione, Michela Gambale, Maristella Berta, Lorena A. Rivera, Amala Mahadevan, Leo Middleton, Francesco M. Falcieri, Stefano Aliani, Annalisa Griffa

**Affiliations:** † 124224 Institute of Marine Sciences, National Research Council (CNR-ISMAR), Venice 30122, Italy; ‡ Monterey Bay Aquarium Research Institute, Moss Landing, California 95039, United States; § Institute of Marine Sciences, National Research Council (CNR-ISMAR), Lerici 19032, Italy; ∥ Mediterranean Institute of Oceanology (MIO), Université de Toulon, La Garde 83130, France; ⊥ PhD Program in Environmental Sciences, Mention in Continental Aquatic System, 28056 University of Concepción, Concepción 4030000, Chile; # 10627 Woods Hole Oceanographic Institution, Woods Hole, Massachusetts 02543, United States

**Keywords:** microfibers retention, submesoscale cyclone, marine contaminants, biological hotspot, Mediterranean Sea, microplastics

## Abstract

Cyclonic eddies are widespread upper ocean features known to enhance primary productivity via nutrient upwelling; yet, their role in the transport and retention of anthropogenic contaminants remains poorly understood. Here, we present high-resolution oceanographic measurements from a submesoscale cyclone in the Western Mediterranean Sea, revealing a pronounced subsurface accumulation of textile microfibers (MFs) within the eddy core (0.34 MF l^–1^) relative to surrounding waters (0.09 MF l^–1^). This enrichment persisted in a secondary, smaller cyclone that detaches following the main cyclone’s fragmentation. Elevated chlorophyll-a concentrations in the upper 40 m, driven by isopycnal uplift, point to a coupled biological response to physical forcing. Spatial heterogeneity in pollution sources, vertical circulation, and mixing likely explains the observed microfiber distribution. Our findings demonstrate that submesoscale cyclones can function as transient yet efficient reservoirs of man-made contaminants, with potential consequences for pollutant exposure pathways and trophic transfer in marine ecosystems.

## Graphical abstract

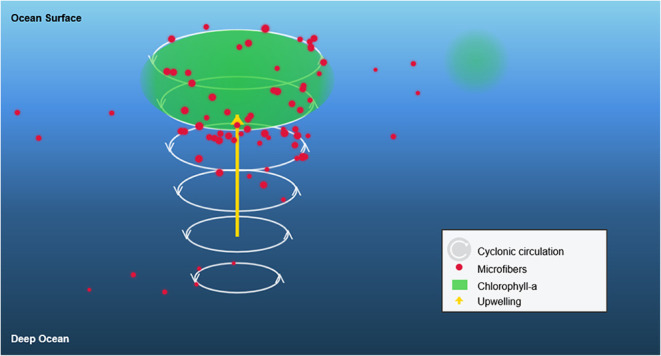

## Introduction

Anthropogenic contaminants such as microplastics and microfibers (MFs) pervade the global ocean, dispersed by a variety of physical processes that operate across spatial and temporal scales.^1^ Studies have well-documented their accumulation in large-scale oceanographic structures like subtropical gyres.^2,3^ In contrast, much less is known about how these contaminants distribute within smaller, ephemeral features, such as submesoscale eddies.

Eddies, ranging from mesoscale (30–100 km; lifespans of weeks to months) to submesoscale (1–10 km; lifespans of hours to days), are ubiquitous features in the global ocean.^4^ Although submesoscale eddies are transient, they play a key role in material transport, vertical nutrient fluxes, and biogeochemical interactions in pelagic ecosystems.^5–9^ In the Northern Hemisphere, cyclonic eddies rotate counterclockwise, and anticyclonic eddies rotate clockwise. Researchers have traditionally associated cyclonic eddies with upwelling and outward particle flux,^10^ although recent observations suggest that cyclonic structures may also retain buoyant particles such as microplastics.^11,12^ Cyclonic eddies can also stimulate biological production by uplifting nutrient-rich waters into the photic zone, fueling phytoplankton blooms and supporting marine food webs.^8,10,13^ Such processes are especially relevant in oligotrophic regions like the offshore Western Mediterranean, where primary production depends heavily on episodic nutrient inputs.^14,15^

The Balearic Sea, located between the Gulf of Lyon and the southwestern Mediterranean, features a complex thermohaline circulation shaped by atmospheric forcing, bathymetry, and frontal dynamics. This region hosts recurrent submesoscale vortices, providing a natural laboratory to study fine-scale ocean transport processes.^16–19^ Eddies generate coherent vorticity fields that often trap particles within their cores,^11,22^ while simultaneously enhancing mixing and dispersion along their peripheries.^23,24^ These advection-diffusion dynamics^20^ are further modulated by vertical motions and wind-driven Ekman transport, especially at density fronts,^21,25^ where convergence zones may foster particle accumulation.

MFs, a dominant subset of marine microplastics,^26–32^ predominantly originate from textile shedding during laundering and regular use. They enter the marine system through atmospheric fallout, wastewater effluents, land runoff, and riverine discharge.^33–36^ Although typically concentrated near coastal areas and at the ocean surface, studies have also detected MFs in deep waters and marine sediments, particularly in the Mediterranean Sea.^37–42^ The transport and vertical distribution of MFs depend on a complex interplay of fiber shape, orientation, turbulence, density stratification, and hydrodynamic patterns.^43–48^ However, we still lack a clear understanding of how energetic submesoscale features influence their sinking, accumulation, and retention.^49–51^

While recent works have highlighted surface accumulation of buoyant plastics in flow convergence zones across multiple scales,^52–55^ few have investigated the subsurface distribution of MFs in actively evolving mesoscale and submesoscale eddies.^30,56–58^ This represents a critical knowledge gap. MFs not only serve as substrates for microbial colonization but are also frequently ingested by plankton and other marine organisms.^33,59–63^ If cyclonic eddies retain MFs while simultaneously acting as biological hotspots, they may facilitate the rapid incorporation of pollutants into marine food webs. Understanding these dynamics is crucial for assessing the fate, transport, and ecological impacts of MF contamination in marine ecosystems.

This study investigates the distribution of MFs within a submesoscale cyclonic eddy in the Balearic Sea, sampled during the 2022 Coherent Lagrangian Pathways from the Surface Ocean to Interior (CALYPSO) campaign (Figure 1). High-resolution oceanographic transects captured the evolution of a cyclonic eddy as it split into two smaller coherent structures.^64^ We focus on two of these transects: one crossing the primary cyclone prior to fragmentation (Figure 1b) and a second crossing the southern smaller cyclone that formed after the split (Figure 1d and Table 1). Subsurface MF accumulation was detected in both eddies and identified through physical (temperature, salinity, current direction) and biogeochemical (dissolved oxygen, chlorophyll-a) variables. We explore the mechanisms driving MF retention and discuss their interaction with marine debris and primary production in submesoscale oceanographic settings.

**Table 1:** Eddy Characteristics^a^

Eddy parameters	Before splitting	After splitting
Major axis (km)	22.22	7.90
Minor axis (km)	9.28	5.39
Area (km^2^)	162.03	33.46
Perimeter (km)	52.82	21.25
Eccentricity	0.91	0.73
Velocity upper 100 m (cm s^–1^)	17.86	18.28

a Eddy boundaries were identified using chlorophyll-a estimates from Figure 1, and horizontal current velocities were derived from Acoustic Doppler Current Profiler data collected during the cruise.

**Figure 1:**
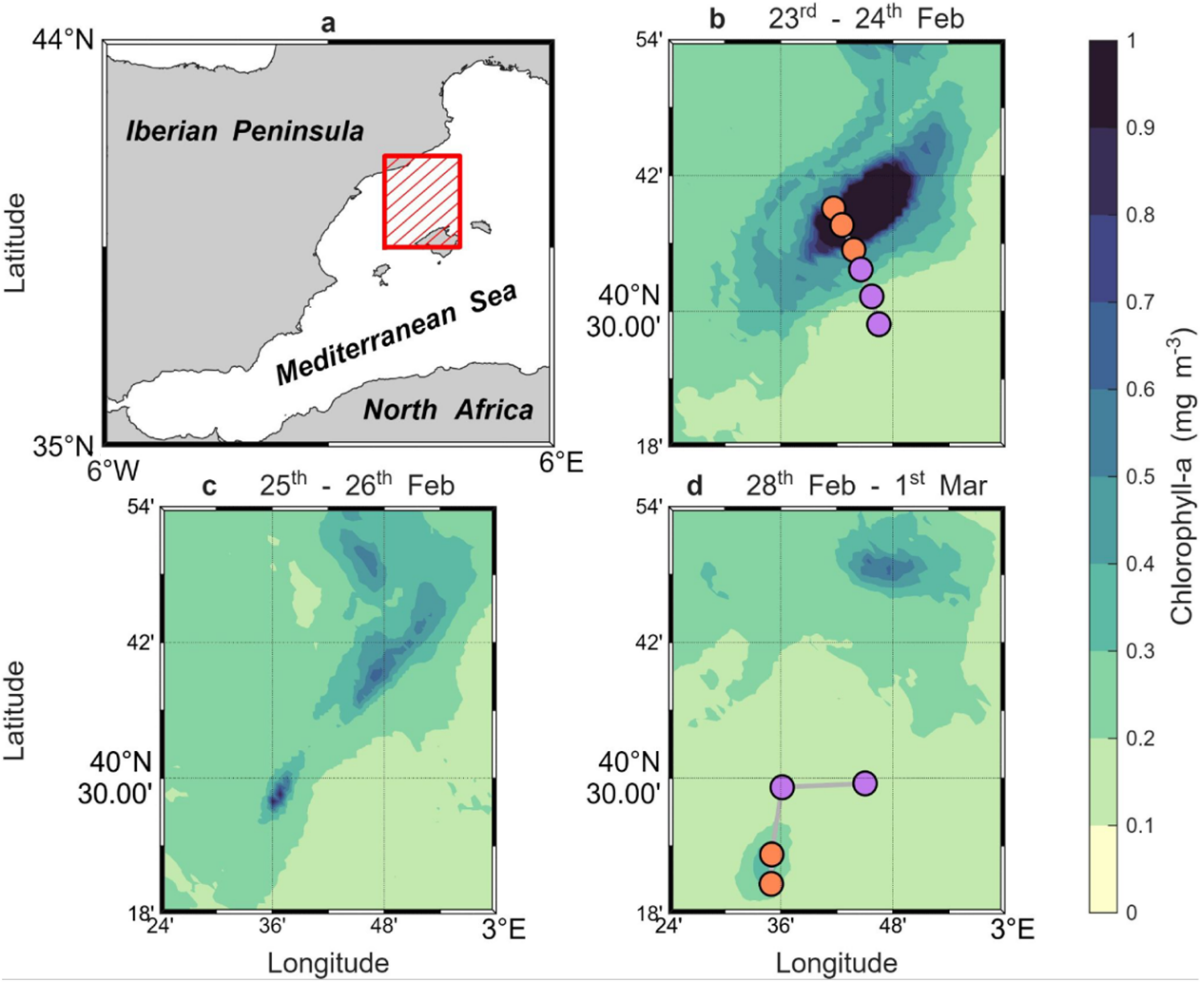
Submesoscale cyclonic eddy in the Western Mediterranean, sampled before and after splitting. (a) Study area in the Balearic Sea. (b–d) Evolution of the eddy splitting pattern observed through satellite chlorophyll-a estimates during the 2022 CALYPSO campaign. The geographic locations of the stations along the two transects are displayed, with the orange (purple) markers indicating stations located inside (outside) the cyclone. Daily Level 4 chlorophyll-a estimates, with a spatial resolution of 1 km, were obtained from the Copernicus Marine Data Store (https://data.marine.copernicus.eu/).

## Materials and Methods

### Sampling Strategy

Sampling was conducted during the CALYPSO 2022 cruise onboard the R/V Pourquoi Pas?, operating in the Balearic Sea between the Iberian Peninsula and the Balearic Islands in the northwest Mediterranean Sea (Figure 1). The campaign aimed to identify subduction pathways from the surface to the ocean interior and assess their influence on the physical and biogeochemical properties. Microfiber (MF) sampling was performed along predefined transects, resulting in 91 water column samples collected by using a Rosette sampler. Conductivity Temperature Depth (CTD) casts typically extended to depths of 200–300 m, with 5–6 sampling depths per station selected based on the CTD down-cast profiles (Supplementary Table 1). At each depth, two 12-l Niskin bottles were generally triggered. Initially, duplicate samples were collected at each depth to account for within-site and between-sample variability.^38^ This included all six stations along the first transect across the main cyclone, with 3 stations inside and 3 outside (Figure 1b). However, due to constraints on the water budget and prolonged filtration times, sampling was reduced to single replicates per depth after February 25th. Consequently, the four stations comprising the second transect across the smaller southern cyclone formed after the eddy splitting (with 2 stations inside and 2 outside; Figure 1d) were collected as single samples (one sample per depth). Despite this adjustment, replicate samples from the first transect showed acceptable agreement and no systematic bias between measurements,^65^ with an absolute mean difference between replicates of 0.02 ± 0.33 MF l^–1^ (mean ± standard deviation; Figure S1).

### Microfiber Quantification

To minimize external contamination, water samples were filtered on the ship’s deck using a closed-loop filtration system that prevented air exposure of the samples throughout the entire sampling procedure. After retrieval of the rosette, a 47 mm stainless-steel filter holder (Advantec MFS, Inc.) was connected to the Niskin bottle faucets using silicone tubing. Filtration was performed using a diaphragm vacuum pump (Rocker Alligator 200) that was directly connected to the filter holder outlet. The filtered volume (median: 10.5 L per sample) was measured using graduated cylinders collecting the water outflow from the pump.

Strict contamination control protocols were enforced throughout the cruise. All sampling equipment was prewashed with filtered water prior to use. Filters and containers were kept covered during sample processing, and the air exposure of sampling equipment was minimized. Procedural blanks were performed at every station by filtering distilled water (prefiltered through GF/D filter cartridges, 2.7 μm pore size) using the same setup and volume as those used for seawater samples. All seawater samples (*n* = 91) and procedural blanks (*n* = 45) were filtered using 47 mm mixed cellulose ester (MCE) membranes with a nominal pore size of 5 μm. Filters were labeled and stored at −20 °C in glass Petri dishes until laboratory analysis. In the laboratory, filters were examined under a stereomicroscope (Leica MZ16) at ×45 magnification. Anthropogenic fibers were identified based on their uniform thickness, absence of cellular structure, coloration, and high tensile strength.^66,67^ MF concentrations were expressed as fibers per liter (MF l^–1^). Procedural blanks revealed minimal contamination during sampling, with fibers detected in 66.7% of the blanks at a median concentration of 0.08 MF l^–1^, significantly lower than environmental values (Mann–Whitney U test, *p* = 1.6 × 10^–11^). All MF concentrations were corrected for procedural blank contamination (Supplementary Table 1), and all negative corrected values were set to zero.

For polymeric composition analysis, 34 seawater samples and 4 procedural blanks were selected for μFTIR spectroscopy. For the stations along the transect in the main eddy, one of the two replicates collected at each depth was randomly selected, and 100% of the recovered MFs (*n* = 171) were analyzed. Fibers were manually extracted, mounted on moistened glass slides, and oven-dried at 40 °C before spectral acquisition. μFTIR analysis was performed using a LUMOS stand-alone FTIR microscope (Bruker Optik GmbH) operated in Attenuated Total Reflectance (ATR) mode.^39^ Fiber dimensions (length and diameter) were measured to the nearest μm by using the digital images captured by the instrument. Spectral data were processed using OPUS 7.5 software, with polymer matches ≥75–80% similarity to reference spectra considered valid. Polymer identification was carried out using a combination of commercially available reference libraries and a custom spectral library developed within the JPI Oceans project BASEMAN.^68^ To enhance identification accuracy, FTIR spectra of common natural and synthetic fabrics, clothing, and textiles were additionally acquired and incorporated into the database based on their label specifications. Fibers were classified as synthetic (e.g., polyester, polypropylene, acrylic), animal-based (e.g., wool), or cellulosic (natural: e.g., cotton; or man-made: e.g., rayon/viscose). Fiber dimensions (length and diameter) and polymer types were consistent across stations and depths. Median length was 539 μm (Q1–Q3: 339–1007 μm), and median diameter was 15 μm (Q1–Q3: 13–19 μm), with no significant differences detected by ANOVA or Kruskal–Wallis^69^ tests. Spearman correlations between station or depth and fiber dimensions were weak and nonsignificant (ρ ≤ 0.19, *p* > 0.01 after correction). Polymer composition was consistently dominated by cellulosic fibers (91.7%; Supplementary Table 2), with minor contributions from synthetic (4.8%) and animal-based fibers (3.4%), and showed no association with station or depth (chi-square, *p* > 0.05). All fibers found in procedural blanks were classified as cellulosic, and no significant differences in length or diameter were observed between fibers recovered from seawater samples and those found in procedural blanks.

### Physical and Biogeochemical Variables

Temperature and salinity were measured with a SeaBird 911plus CTD probe. Following postprocessing, the data were binned into 1-m intervals. CTD-derived chlorophyll-a fluorescence (WETStar Fluorometer) was calibrated against *in situ* chlorophyll-a concentrations measured fluorometrically.^70^ The SeaBird 43 dissolved oxygen sensor was calibrated before and after the campaign and showed no significant drift. Conservative temperature, absolute salinity, and potential density were calculated using the Gibbs SeaWater (GSW) oceanographic toolbox (https://www.teos-10.org/software.htm). Current velocity and direction were measured by using a hull-mounted 150 kHz Teledyne RDI Ocean Surveyor Acoustic Doppler Current Profiler (ADCP), operating continuously during the cruise, with a vertical bin size of 4 m (Figure S2).

### Clustering of Oceanographic Stations along the Transects

K-means nonhierarchical clustering was used to group stations based on similarities in upper-layer hydrography.^71^ The mean values of temperature, salinity, oxygen, and chlorophyll-a within the upper 50 m were used as clustering variables. Data were normalized to enhance the algorithm’s performance,^72^ followed by clustering using the Euclidean distance metric. The optimal number of clusters (*k* = 2) was determined using silhouette scores after 1000 iterations, yielding a total within-cluster sum of distances of 1.09 (Figure S3). Stations from the main and smaller eddies clustered together, while the remaining five external stations formed a separate group (Figure 1). Hierarchical clustering using the farthest distance method confirmed this division. A dendrogram with a cophenetic correlation coefficient of 0.65 was generated (Figure S3) based on the degree of dissimilarity of physical and biogeochemical oceanographic properties. Both clustering methods revealed a grouping pattern consistent with patterns observed in satellite chlorophyll-a imagery (Figure 1) and vertical CTD profiles (Figure 2 and Figure S4). Mean values of physical and biogeochemical variables across several depth ranges (0–75, 100, 150, and 200 m) were also analyzed using cluster analysis. The results indicated a consistent division of stations except for variability attributed to the third station located within the smaller cyclone formed after the splitting event.

**Figure 2:**
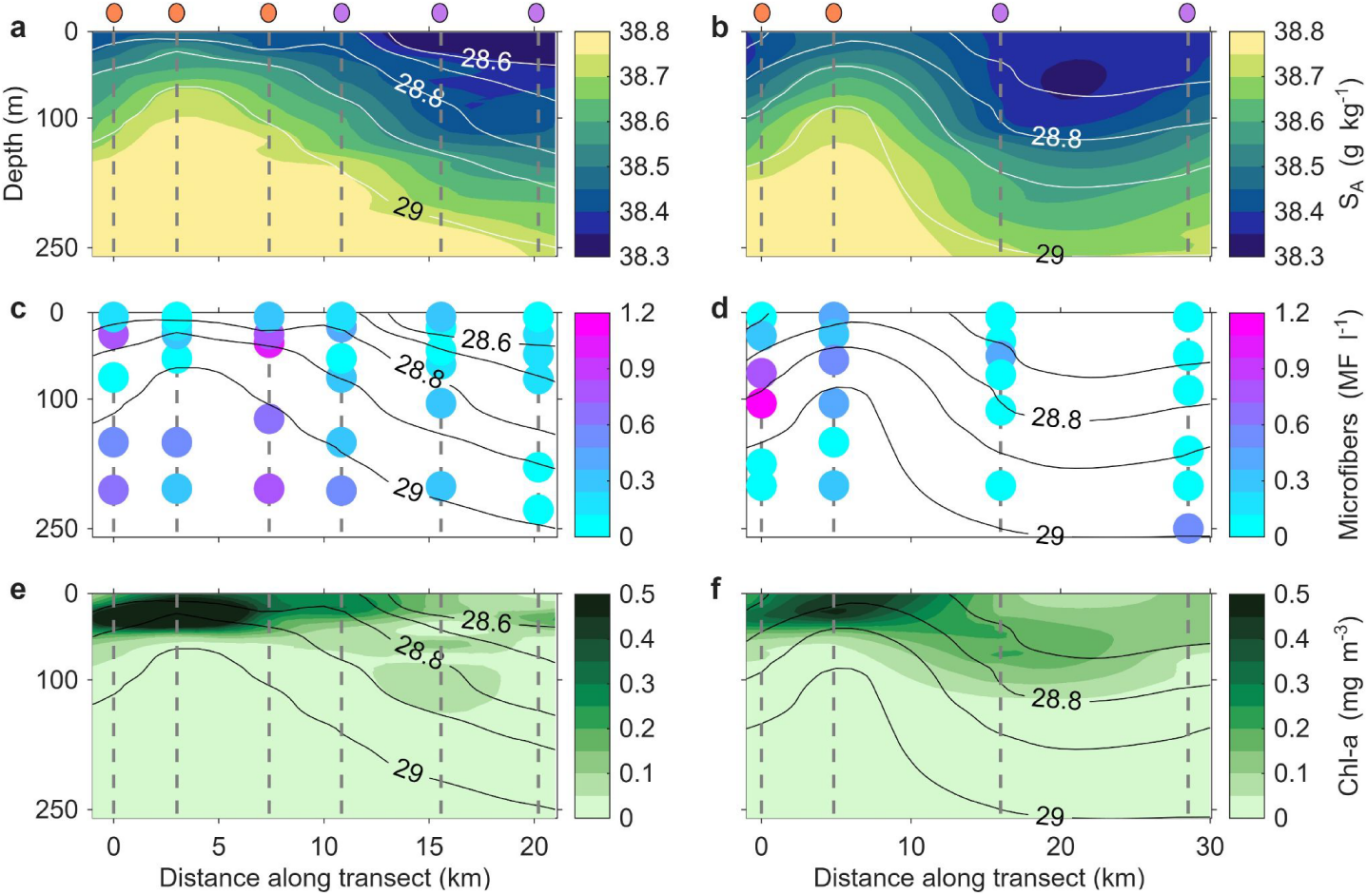
Profiles of salinity, microfiber concentrations, and chlorophyll-a along the two transects. (a, b) Absolute salinity. (c, d) Microfiber concentration. (e, f) Chlorophyll-a concentration. The left (right) panels show the transects in the eddy before (after) the split (Figure 1). Contours represent potential density anomaly. Water sampling locations are indicated by gray dashed lines, with colored markers above the upper panels denoting whether stations were located inside (orange) or outside (purple) the cyclone. Absolute salinity and chlorophyll-a concentration were measured using a Conductivity Temperature Depth (CTD) profiler. Note: color scales for microfiber panels (c, d) are set to 0–1.2 MF l^–1^ to optimize visualization of spatial patterns. The complete data range is shown in Figure 3.

### Statistical Differences in MFs Collected Inside and Outside the Eddy

The distribution of MF concentration data was assessed using the Kolmogorov–Smirnov test,^73^ which indicated an absence of normality (*p* = 1.9 × 10^–13^). Consequently, a nonparametric Mann–Whitney U-test^74^ was used to evaluate differences between MF concentrations measured inside and outside the eddy, as well as between procedural blanks and seawater samples.

## Results and Discussion

### Cyclone Detection and Characterization

Cyclonic eddies were identified along both transects based on the combined analysis of salinity, density, and chlorophyll-a profiles (Figure 2) and further supported by temperature, dissolved oxygen, and current direction patterns (Figure S4). A prominent uplift of isopycnals was observed near the eddy core, with the greatest vertical displacement occurring around a 100 m depth. At this depth, saline, low-oxygen, and chlorophyll-a-depleted waters were brought upward from deep layers. Current velocities derived from ADCP data revealed counter-rotating flow across the eddy center, consistent with the characteristic anticlockwise circulation of Northern Hemisphere cyclones.

Outside the eddy, isopycnals remained flat, and water masses exhibited distinct physical and biogeochemical properties. Cyclone boundaries were delineated using hydrological criteria (detailed in Materials and Methods), enabling the classification of sampling stations as either located “inside” or “outside” the eddies.

Mean chlorophyll-a concentrations in the upper 40 m were significantly higher inside the eddy (0.44 mg m^–3^) than outside (0.15 mg m^–3^), as shown in Figure 2 and Figure S5. Dissolved oxygen levels followed a similar trend, averaging 8.0 ml l^–1^ within the cyclone compared to 7.7 ml l^–1^ outside. Both differences were statistically significant (Mann–Whitney U test, *p* < 0.05), consistent with elevated phytoplankton biomass within the eddy.

### Microfiber Accumulation in Submesoscale Cyclones

A total of 1182 MFs were counted in the water samples, yielding a median concentration of 0.17 MF l^–1^ across the entire data set (interquartile range Q1–Q3: 0.08–0.33 MF l^–1^). Consistent with previous findings,^39^ the majority of MFs (91.7%) were identified as cellulosic (Supplementary Table 2), followed by synthetic polymers and animal-based fibers, indicating primarily negatively buoyant polymers. The polymeric composition did not differ significantly between samples collected inside and outside the cyclone.

MF concentrations were significantly higher within the eddy cores (*p* < 0.05), particularly at the first and third stations of the first transect and the first station of the second transect (Figure 2). When data from both transects were merged, median MF concentrations inside the eddy reached 0.34 MF l^–1^, nearly four times higher than outside (0.09 MF l^–1^, *p* = 3.7 × 10^–4^). Analyzed separately, median concentrations inside the eddy before its fragmentation reached 0.44 MF l^–1^, compared to 0.19 MF l^–1^ outside. After the eddy split, values remained elevated within the smaller southern eddy (0.32 MF l^–1^), while concentrations outside dropped to 0.01 MF l^–1^ (*p* = 0.01 in both cases; Figure 3). These results indicate a 27% decline in MF concentration within the eddy core postfragmentation and a striking 95% reduction in surrounding waters, underscoring the cyclone’s role as a transient but efficient MF retention structure. Within the eddy, MF concentrations exhibited clear vertical and horizontal gradients, with a subsurface maximum typically observed along the doming of the 28.9 kg m^–3^ isopycnal. Concentrations were generally higher along the flanks than at the eddy center (median = 0.58 MF l^–1^ vs 0.32 MF l^–1^, *p* = 0.07), suggesting lateral convergence and retention near the periphery. In contrast, outside the eddy, MF profiles were vertically homogeneous and consistently low, reflecting the absence of strong recirculating flow and vertical transport.

**Figure 3:**
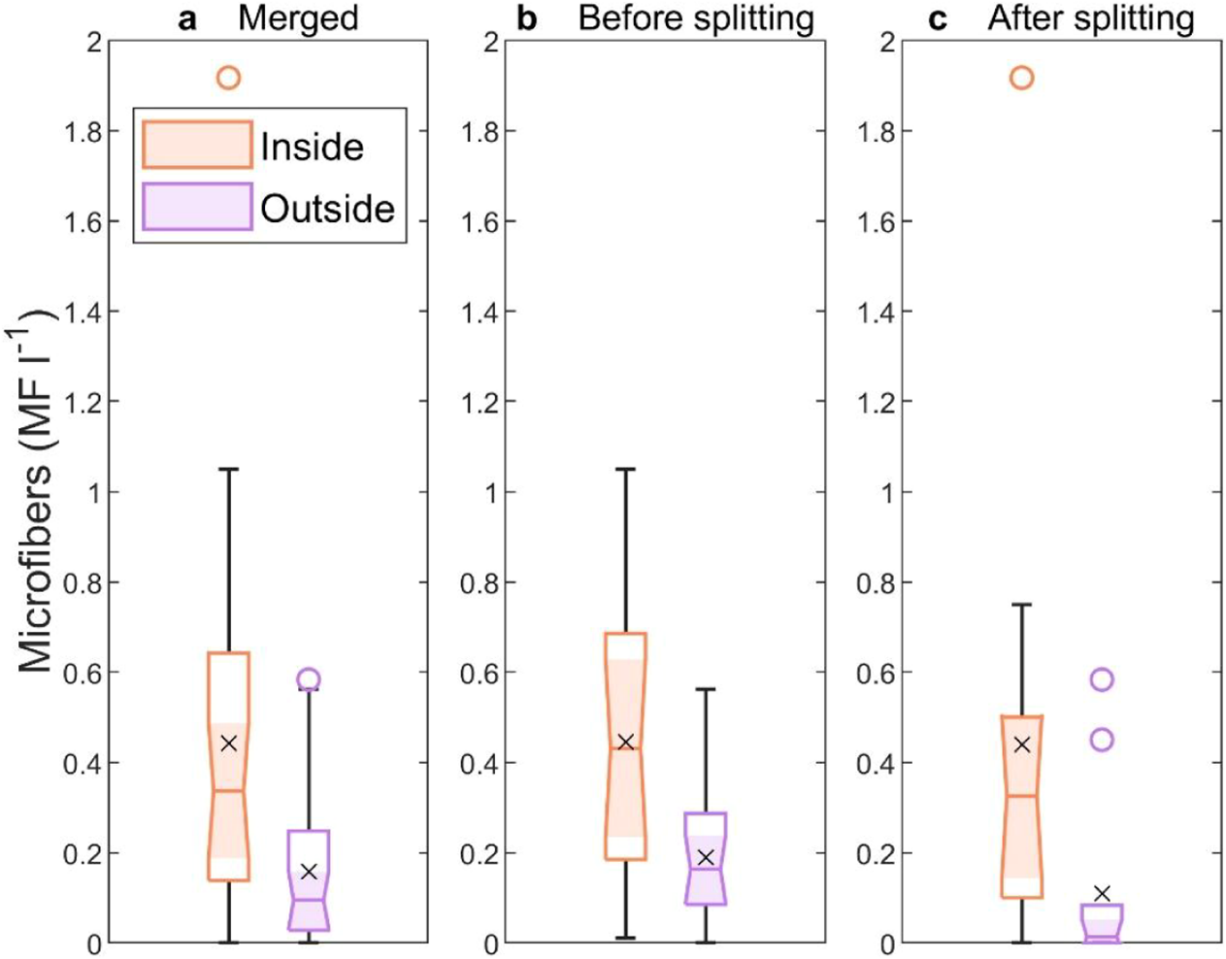
Microfiber concentrations measured inside and outside the cyclones. (a) Combined data from both transects. (b) Data from the transect conducted before the eddy splitting. (c) Data from the transect conducted after the eddy splitting. The median MF concentration is represented by a line inside each box, while the mean is indicated by an “*x*”. Outliers, computed using the interquartile range, are displayed as circular markers. The lower and upper quartiles are represented by the bottom and top edges of the box, respectively. Whiskers extend to the maximum and minimum values, excluding outliers. Orange and purple represent data collected inside and outside the cyclones, respectively.

When compared with published data sets, the MF concentrations observed in this study are substantially lower than those typically reported for Mediterranean and open-ocean waters.^29,30,39^ Even the highest values within the cyclone core (0.58 MF l^–1^) are about an order of magnitude below previously published ranges (2–8 MF l^–1^). This difference primarily reflects our use of a closed-loop filtration system that eliminated external contamination, thereby producing more conservative and environmentally representative estimates. Despite these lower absolute concentrations, the 4-fold enrichment observed inside the cyclone relative to surrounding waters confirms its function as a transient retention “hotspot” for microfibers.

Although the highest MF concentrations were observed near the eddy edge, statistical comparisons between edge and core values (*p* = 0.07) indicate that this difference was not significant. Given the limited number of sampling stations within the eddy, particularly along the second transect, these patterns should be interpreted cautiously.

### Mechanisms Driving Microfiber Accumulation

The cyclone formed along the Balearic front, a persistent mesoscale boundary separating denser, cyclonic waters in the northwest from lighter, anticyclonic waters in the southeast (Figure S6).^75^ Although sinking particles typically settle within a few tens of kilometers from their source,^76,77^ recent studies have shown that mesoscale circulation and frontal dynamics in the Balearic Sea can transport microplastics and other anthropogenic contaminants from coastal areas toward offshore regions.^18,41,47^ This regional connectivity may partly explain the MF heterogeneity across the front (Figure S7), with denser, northwest-sourced waters potentially carrying elevated MF loads due to their coastal provenance. Nevertheless, water mass characteristics and source proximity alone cannot fully explain the MF enrichment observed within the cyclone. The strong vertical and horizontal gradients are best explained by physical retention processes, consistent with the local hydrographic structure and circulation patterns. We therefore focus on the cyclone’s internal physical dynamics as the primary drivers of MF retention, assuming a uniform MF input dominated by surface atmospheric deposition.^34,35,78^

The vertical settling behavior of rigid microplastic particles has been extensively studied in laboratory settings.^79–81^ Under quiescent conditions, negatively buoyant particles, such as MFs, sink at a constant settling rate (*ws*) determined by their size, shape, and density. The Maxey–Riley–Gatignol (MRG) equation^82^ provides a theoretical framework to quantify *ws* for small, near-spherical particles.^77,83^ However, the actual particle velocity (*wp*) combines *ws* with the ambient fluid velocity (*w*), and determining *wp* is complex due to MFs’ flexibility and pronounced deviation from sphericity.^45,84^ Consequently, simplified formulas for *ws* are often unreliable, and even the concept of settling velocity becomes ambiguous.^44^ Laboratory studies report settling velocities of 0.5–3.7 mm s^–1^ in still water for synthetic MFs.^44,45^ However, in natural systems, convective and turbulent motions can substantially reduce the effective sedimentation flux by generating upward and oscillatory flows that resuspend or vertically redistribute fibers, keeping them suspended for extended periods. Such processes can decrease the total downward flux by up to 75%, effectively lowering the apparent sedimentation rate without altering the intrinsic settling velocity of the particles.^77,85,86^

Vertical velocities in the ocean vary by scale: small-scale processes such as Langmuir cells and boundary layer turbulence can generate velocities of several cm s^–1^ near the surface,^85,86^ while mesoscale structures exhibit lower velocities, typically up to 1 mm s^–1^.^87,88^ Drifter-derived measurements along the cyclone flank before the splitting indicate upward vertical velocities of 0.7 mm s^–1^ within the upper 15 m.^89^ This provides quantitative evidence of the presence of submesoscale processes capable of significant vertical transport, therefore impacting the MF settling velocity. Indeed, vertical velocities within the cyclone are likely due to several interacting processes. On the one hand, we expect a general upwelling pattern within the eddy core, as indicated by the domed isopycnals, subsurface oxygen depletion, and elevated surface chlorophyll-a concentrations (Figure 2 and Figure S4). On the other hand, more transient processes are likely to generate significant vertical recirculation.^25^ These include alternating cyclogeostrophic upwelling/downwelling associated with the cyclone’s elongated structure and possible distortion,^64,90^ possible wind-induced Ekman pumping processes,^89^ and near-inertial oscillations.^88^ Also, vertical mixing at the front could lead to turbulent thermal wind balance (TTW), driving secondary circulations with enhanced vertical velocity.^91,92^

Overall, the presence of these motions likely retains negatively buoyant MFs in suspension, explaining their observed presence in the cyclone.^39^ Although the higher density of water within the cyclone may slightly reduce MF settling compared to adjacent waters (Figures S5-S6), such effects are likely negligible relative to the dynamical processes discussed above.^77^ We note that TTW processes occurring at the fronts might contribute to MF accumulation at eddy edges, as well as the interaction between the cyclone’s dynamic structure and particle movements under asymmetric and nonsteady flow conditions.^93^ While this is consistent with the qualitative pattern shown by the MF concentration in the cyclone (Figure 2), we recall that the difference between the edges and the core is not statistically significant and could be due to limited sampling and transient processes. Indeed, the measurements are not sufficient to sort out the specific effects of each dynamical mechanism on MF resuspension. The combination of elevated chlorophyll-a, domed isopycnals, and counter-rotating currents at the eddy center nonetheless supports the interpretation that the cyclone acted as a coherent retention structure for microfibers, even if local concentrations varied spatially within its interior.

Further insight into MF retention could be achieved by applying idealized vortex models or high-resolution simulations nested within regional forecasting systems.^94^ However, developing suitable particle models for MFs remains a key challenge, as conventional approaches like the MRG equation fail to capture the behavior of dense, flexible fibers without incorporating semiempirical corrections and fiber-specific hydrodynamic properties.^44,95,96^

### Ecological Implications of Microfiber Retention in Eddies

Our high-resolution observations underscore the dual role of submesoscale eddies as zones of both enhanced biological productivity and pollutant retention in pelagic ecosystems. The co-occurrence of elevated chlorophyll-a and MF concentrations within the cyclone reveals a critical dynamic-ecological coupling: the physical processes that retain negatively buoyant MFs (i.e., upwelling, vertical recirculation, and frontal convergence) simultaneously enhance nutrient supply and primary production. This may create conditions where phytoplankton blooms develop in contaminant-enriched waters, potentially facilitating rapid MF incorporation into marine food webs.^97^ Vertical retention of negatively buoyant MFs within the photic zone keeps them bioavailable and increases encounter rates with plankton, filter feeders, and planktivorous organisms.^98^ Their widespread detection in zooplankton, commercially important fish species, seabirds, and marine mammals, reflects the pervasiveness of microfiber contamination, which often dominates the spectrum of ingested anthropogenic particles.^33,99–101^ While species-specific toxicological implications remain uncertain,^60,102–105^ the potential for bioaccumulation, trophic transfer, and broader ecological ramifications raises significant concerns.^32^

Despite compelling evidence of MF retention within submesoscale cyclones, our study has several limitations. Observations were limited to two transects over a short temporal window, precluding a full reconstruction of the eddy’s three-dimensional structure, temporal evolution, and retention timescales. Vertical velocities were inferred from isopycnal displacement rather than measured directly, and although the methodology was kept consistent for all samples, MF quantification may be subject to sampling uncertainty and operator classification bias. The biological response variables were limited to chlorophyll-a concentrations; direct measurements of zooplankton abundance, feeding rates, or MF ingestion within the eddy were not obtained and would strengthen future investigations of dynamic-ecological coupling. Moreover, the specific hydrographic context of the Balearic Sea may constrain a broader extrapolation of our findings.

Nonetheless, our results are consistent with previous reports identifying hotspots of microplastics-marine life interactions in other small-scale surface ocean features, such as fronts and convergence zones.^106^ Although transient and small in scale, submesoscale eddies may act similarly, functioning as dynamic and biologically significant retention structures in pelagic ecosystems. Given their ubiquity across global oceans, these features likely play a more significant role in contaminant redistribution than those previously recognized. This study bridges a critical gap between physical oceanography and marine pollution science by providing direct field evidence of microfiber accumulation in submesoscale eddies. Integrating these dynamics into contaminant transport models is essential to improving predictions of microplastic and microfiber fate in the ocean. Future work should focus on quantifying retention times, assessing seasonal and regional variability in eddy-driven MF accumulation, and evaluating biological uptake within these widespread dynamic features.

## Supplementary Material



## Notes

Additional tables and supporting figures, including the Bland–Altman plot (Figure S1), sampling locations for ADCP and CTD-Niskin (Figure S2), clustering of oceanographic stations (Figure S3), vertical profiles of physical and biogeochemical properties (Figures S4–S5), density front locations (Figure S6), TS diagrams (Figure S7), microfiber concentrations before and after blank correction (Table S1), and microfiber composition (Table S2); microfiber data provided in Table S1, while CTD and ADCP data available from the Woods Hole Open Access Server (10.26025/1912/71856) (PDF)

## References

[1] van
Sebille E., Aliani S., Law K. L., Maximenko N., Alsina J. M., Bagaev A., Bergmann M., Chapron B., Chubarenko I., Cózar A., Delandmeter P., Egger M., Fox-Kemper B., Garaba S. P., Goddijn-Murphy L., Hardesty B. D., Hoffman M. J., Isobe A., Jongedijk C. E., Kaandorp M. L. A., Khatmullina L., Koelmans A. A., Kukulka T., Laufkötter C., Lebreton L., Lobelle D., Maes C., Martinez-Vicente V., Morales Maqueda M. A., Poulain-Zarcos M., Rodríguez E., Ryan P. G., Shanks A. L., Shim W. J., Suaria G., Thiel M., Van Den Bremer T. S., Wichmann D. (2020). The Physical Oceanography of the Transport of Floating
Marine Debris. Environ. Res. Lett..

[2] Courtene-Jones W., van Gennip S., Penicaud J., Penn E., Thompson R. C. (2022). Synthetic
Microplastic Abundance and Composition along a Longitudinal Gradient
Traversing the Subtropical Gyre in the North Atlantic Ocean. Mar. Pollut. Bull..

[3] Lebreton L., Slat B., Ferrari F., Sainte-Rose B., Aitken J., Marthouse R., Hajbane S., Cunsolo S., Schwarz A., Levivier A. (2018). Evidence That the Great
Pacific Garbage Patch Is Rapidly Accumulating Plastic. Sci. Rep..

[4] Chen G., Han G. (2019). Contrasting Short-Lived
With Long-Lived Mesoscale Eddies in the Global
Ocean. J. Geophys. Res.: Oceans.

[5] Chelton D. B., Schlax M. G., Samelson R. M. (2011). Global Observations of Nonlinear
Mesoscale Eddies. Prog. Oceanogr..

[6] Early J. J., Samelson R. M., Chelton D. B. (2011). The Evolution
and Propagation of
Quasigeostrophic Ocean Eddies*. J. Phys. Oceanogr..

[7] McGillicuddy D. J. (2016). Mechanisms
of Physical-Biological-Biogeochemical Interaction at the Oceanic Mesoscale. Ann. Rev. Mar. Sci..

[8] Belkin N., Guy-Haim T., Rubin-Blum M., Lazar A., Sisma-Ventura G., Kiko R., Morov A. R., Ozer T., Gertman I., Herut B., Rahav E. (2022). Influence
of Cyclonic and Anticyclonic
Eddies on Plankton in the Southeastern Mediterranean Sea during Late
Summertime. Ocean Sci..

[9] Lévy M., Couespel D., Haëck C., Keerthi M. G., Mangolte I., Prend C. J. (2024). The Impact of Fine-Scale
Currents on Biogeochemical
Cycles in a Changing Ocean. Ann. Rev. Mar. Sci..

[10] Klein P., Lapeyre G. (2009). The Oceanic Vertical
Pump Induced by Mesoscale and
Submesoscale Turbulence. Ann. Rev. Mar. Sci..

[11] Haller G., Beron-Vera F. J. (2013). Coherent
Lagrangian Vortices: The Black Holes of Turbulence. J. Fluid Mech..

[12] Beron-Vera F. J., Olascoaga M. J., Lumpkin R. (2016). Inertia-Induced Accumulation of Flotsam
in the Subtropical Gyres. Geophys. Res. Lett..

[13] McGillicuddy D. J., Robinson A. R., Siegel D. A., Jannasch H. W., Johnson R., Dickey T. D., McNeil J., Michaels A. F., Knap A. H. (1998). Influence
of Mesoscale Eddies on New Production in the Sargasso Sea. Nature.

[14] Coll M., Piroddi C., Steenbeek J., Kaschner K., Lasram F. B. R., Aguzzi J., Ballesteros E., Bianchi C. N., Corbera J., Dailianis T. (2010). The Biodiversity
of the Mediterranean Sea: Estimates,
Patterns, and Threats. PLoS One.

[15] Browning T. J., Moore C. M. (2023). Global Analysis
of Ocean Phytoplankton Nutrient Limitation
Reveals High Prevalence of Co-Limitation. Nat.
Commun..

[16] Rubio A., Arnau P. A., Espino M., Del Mar Flexas M., Jordà G., Salat J., Puigdefàbregas J., Arcilla A. S. (2005). A Field Study of the Behaviour of an Anticyclonic Eddy
on the Catalan Continental Shelf (NW Mediterranean). Prog. Oceanogr..

[17] Capó E., Orfila A., Mason E., Ruiz S. (2019). Energy Conversion
Routes
in the Western Mediterranean Sea Estimated from Eddy-Mean Flow Interactions. J. Phys. Oceanogr..

[18] Cotroneo Y., Celentano P., Aulicino G., Perilli A., Olita A., Falco P., Sorgente R., Ribotti A., Budillon G., Fusco G., Pessini F. (2021). Connectivity Analysis Applied to
Mesoscale Eddies in the Western Mediterranean Basin. Remote Sens..

[19] Aguiar E., Mourre B., Alvera-Azcárate A., Pascual A., Mason E., Tintoré J. (2022). Strong Long-Lived Anticyclonic Mesoscale
Eddies in the Balearic Sea: Formation, Intensification, and Thermal
Impact. J. Geophys. Res.: Oceans.

[20] Dong C., McWilliams J. C., Liu Y., Chen D. (2014). Global Heat
and Salt
Transports by Eddy Movement. Nat. Commun..

[21] Okubo A. (1971). Oceanic Diffusion
Diagrams. Deep Sea Res. Oceanogr. Abstr..

[22] Barabinot Y., Speich S., Carton X. (2024). Defining Mesoscale Eddies Boundaries
From In-Situ Data and a Theoretical Framework. J. Geophys. Res.: Oceans.

[23] Dritschel D. G., McIntyre M. E. (2008). Multiple Jets as PV Staircases: The Phillips Effect
and the Resilience of Eddy-Transport Barriers. J. Atmos. Sci..

[24] McWilliams J. C. (2016). Submesoscale
Currents in the Ocean. Proc. R. Soc. A.

[25] Mahadevan A. (2016). The Impact
of Submesoscale Physics on Primary Productivity of Plankton. Ann. Rev. Mar. Sci..

[26] Sanchez-Vidal A., Thompson R. C., Canals M., De Haan W. P. (2018). The Imprint of Microfibres
in Southern European Deep Seas. PLoS One.

[27] Gago J., Carretero O., Filgueiras A. V., Viñas L. (2018). Synthetic
Microfibers in the Marine Environment: A Review on Their Occurrence
in Seawater and Sediments. Mar. Pollut. Bull..

[28] Reineccius J., Appelt J. S., Hinrichs T., Kaiser D., Stern J., Prien R. D., Waniek J. J. (2020). Abundance
and Characteristics of
Microfibers Detected in Sediment Trap Material from the Deep Subtropical
North Atlantic Ocean. Sci. Total Environ..

[29] Pedrotti M. L., Petit S., Eyheraguibel B., Kerros M. E., Elineau A., Ghiglione J. F., Loret J. F., Rostan A., Gorsky G. (2021). Pollution
by Anthropogenic Microfibers in North-West Mediterranean Sea and Efficiency
of Microfiber Removal by a Wastewater Treatment Plant. Sci. Total Environ..

[30] Rios-Fuster B., Compa M., Alomar C., Fagiano V., Ventero A., Iglesias M., Deudero S. (2022). Ubiquitous Vertical Distribution
of Microfibers within the Upper Epipelagic Layer of the Western Mediterranean
Sea. Estuar. Coast. Shelf Sci..

[31] Tirelli, V. ; Suaria, G. ; Lusher, A. L. Microplastics in Polar Samples. In Handbook of Microplastics in the Environment. Rocha-Santos, T. ; Costa, M. F. ; Mouneyrac, C. , Eds.; Springer, 2022, pp. 281–322.

[32] Athey S. N., Erdle L. M. (2022). Are We Underestimating Anthropogenic
Microfiber Pollution?
A Critical Review of Occurrence, Methods, and Reporting. Environ. Toxicol. Chem..

[33] Liu J., Liu Q., An L., Wang M., Yang Q., Zhu B., Ding J., Ye C., Xu Y. (2022). Microfiber Pollution
in the Earth System. Rev. Environ. Contam. Toxicol..

[34] Napper I. E., Parker-Jurd F. N. F., Wright S. L., Thompson R. C. (2023). Examining the Release
of Synthetic Microfibres to the Environment via Two Major Pathways:
Atmospheric Deposition and Treated Wastewater Effluent. Sci. Total Environ..

[35] Martynova A., Genchi L., Laptenok S. P., Cusack M., Stenchikov G. L., Liberale C., Duarte C. M. (2024). Atmospheric Microfibrous Deposition
over the Eastern Red Sea Coast. Sci. Total Environ..

[36] Suaria, G. The Occurrence of Natural and Synthetic Fibers in the Marine Environment. In Microfibre Pollution from Textiles; CRC Press, 2024, pp. 245–262.

[37] Desforges J. P. W., Galbraith M., Dangerfield N., Ross P. S. (2014). Widespread Distribution
of Microplastics in Subsurface Seawater in the NE Pacific Ocean. Mar. Pollut. Bull..

[38] Ryan P. G., Suaria G., Perold V., Pierucci A., Bornman T. G., Aliani S. (2020). Sampling Microfibres
at the Sea Surface: The Effects
of Mesh Size, Sample Volume and Water Depth. Environ. Pollut..

[39] Suaria G., Achtypi A., Perold V., Lee J. R., Pierucci A., Bornman T. G., Aliani S., Ryan P. G. (2020). Microfibers in Oceanic
Surface Waters: A Global Characterization. Sci.
Adv..

[40] Suaria, G. ; Musso, M. ; Achtypi, A. ; Bassotto, D. ; Aliani, S. Textile Fibres in Mediterranean Surface Waters: Abundance and Composition. In Proceedings of the 2nd International Conference on Microplastic Pollution in the Mediterranean Sea, Cocca, M. ; Di Pace, E. ; Errico, M. E. ; Gentile, G. ; Montarsolo, A. ; Mossotti, R. ; Avella, M. , Eds.; Springer, 2020, pp. 62–66.

[41] Paluselli, A. ; Suaria, G. ; Borghini, M. ; Vitale, G. ; Aliani, S. Mediterranean Intermediate and Deep Waters as Reservoirs and Carriers of Small Microfibers. In 43RD Ciesm Congress Proceedings; Springer, 2024.

[42] Fagiano V., Compa M., Alomar C., Rios-Fuster B., Morató M., Capó X., Deudero S. (2023). Breaking the Paradigm:
Marine Sediments Hold Two-Fold Microplastics than Sea Surface Waters
and Are Dominated by Fibers. Sci. Total Environ..

[43] Vega-Moreno D., Abaroa-Pérez B., Rein-Loring P. D., Presas-Navarro C., Fraile-Nuez E., Machín F. (2021). Distribution and Transport of Microplastics
in the Upper 1150 m of the Water Column at the Eastern North Atlantic
Subtropical Gyre, Canary Islands, Spain. Sci.
Total Environ..

[44] Khatmullina L., Chubarenko I. (2021). Thin Synthetic Fibers Sinking in Still and Convectively
Mixing Water: Laboratory Experiments and Projection to Oceanic Environment. Environ. Pollut..

[45] Goral K. D., Guler H. G., Larsen B. E., Carstensen S., Christensen E. D., Kerpen N. B., Schlurmann T., Fuhrman D. R. (2023). Settling Velocity of Microplastic Particles Having
Regular and Irregular Shapes. Environ. Res..

[46] Woodall L. C., Sanchez-Vidal A., Canals M., Paterson G. L. J., Coppock R., Sleight V., Calafat A., Rogers A. D., Narayanaswamy B. E., Thompson R. C. (2014). The Deep Sea Is a Major Sink for Microplastic Debris. R. Soc. Open Sci..

[47] Suaria, G. ; Berta, M. ; Griffa, A. ; Molcard, A. ; Özgökmen, T. M. ; Zambianchi, E. ; Aliani, S. Dynamics of Transport, Accumulation, and Export of Plastics at Oceanic Fronts. In Chemical Oceanography of Frontal Zones; The Handbook of Environmental Chemistry, Belkin, I. M. , ed.; Springer, 2021, pp. 355–405.

[48] Zhao S., Kvale K. F., Zhu L., Zettler E. R., Egger M., Mincer T. J., Amaral-Zettler L. A., Lebreton L., Niemann H., Nakajima R., Thiel M., Bos R. P., Galgani L., Stubbins A. (2025). The Distribution of
Subsurface Microplastics in the
Ocean. Nature.

[49] Lindström S. B., Uesaka T. (2007). Simulation of the Motion
of Flexible Fibers in Viscous
Fluid Flow. Phys. Fluids.

[50] Bagaev A., Mizyuk A., Khatmullina L., Isachenko I., Chubarenko I. (2017). Anthropogenic Fibres in the Baltic Sea Water Column:
Field Data, Laboratory and Numerical Testing of Their Motion. Sci. Total Environ..

[51] Du
Roure O., Lindner A., Nazockdast E. N., Shelley M. J. (2019). Dynamics of Flexible Fibers in Viscous Flows and Fluids. Annu. Rev. Fluid Mech..

[52] D’Asaro E. A., Shcherbina A. Y., Klymak J. M., Molemaker J., Novelli G., Guigand C. M., Haza A. C., Haus B. K., Ryan E. H., Jacobs G. A., Huntley H. S., Laxague N. J. M., Chen S., Judt F., McWilliams J. C., Barkan R., Kirwan A. D., Poje A. C., Özgökmen T. M. (2018). Ocean Convergence
and the Dispersion of Flotsam. Proc. Natl. Acad.
Sci. U. S. A..

[53] Brach L., Deixonne P., Bernard M. F., Durand E., Desjean M. C., Perez E., van Sebille E., Ter Halle A. (2018). Anticyclonic
Eddies Increase Accumulation of Microplastic in the North Atlantic
Subtropical Gyre. Mar. Pollut. Bull..

[54] Nakajima R., Nagano A., Osafune S., Tsuchiya M., Fujikura K. (2024). Aggregation
and Transport of Microplastics by a Cold-Core Ring in the Southern
Recirculation of the Kuroshio Extension: The Role of Mesoscale Eddies
on Plastic Debris Distribution. Ocean Dyn..

[55] Capodici F., Corbari L., Gauci A., Basilone G., Bonanno A., Campanella S., Ciraolo G., Candela A., D’Amato D., Ferreri R., Fontana I., Genovese S., Giacalone G., Marino G., Aronica S. (2024). Towards Microplastic Hotspots Detection:
A Comparative Analysis of in-Situ Sampling and Sea Surface Currents
Derived by HF Radars. Mar. Pollut. Bull..

[56] Reisser J., Slat B., Noble K., Du Plessis K., Epp M., Proietti M., De Sonneville J., Becker T., Pattiaratchi C. (2015). The Vertical
Distribution of Buoyant Plastics at Sea: An Observational Study in
the North Atlantic Gyre. Biogeosciences.

[57] Egger M., Schilt B., Wolter H., Mani T., de Vries R., Zettler E., Niemann H. (2022). Pelagic distribution of plastic debris
(> 500 μm) and marine organisms in the upper layer
of
the North Atlantic Ocean. Sci. Rep..

[58] Gunaalan K., Almeda R., Vianello A., Lorenz C., Iordachescu L., Papacharalampos K., Nielsen T. G., Vollertsen J. (2024). Does Water
Column Stratification Influence the Vertical Distribution of Microplastics?. Environ. Pollut..

[59] Desforges J. P. W., Galbraith M., Ross P. S. (2015). Ingestion of Microplastics by Zooplankton
in the Northeast Pacific Ocean. Arch. Environ.
Contam. Toxicol..

[60] Kang J. H., Kwon O. Y., Hong S. H., Shim W. J. (2020). Can Zooplankton
Be Entangled by Microfibers in the Marine Environment?: Laboratory
Studies. Water.

[61] Wright R. J., Erni-Cassola G., Zadjelovic V., Latva M., Christie-Oleza J. A. (2020). Marine
Plastic Debris: A New Surface for Microbial Colonization. Environ. Sci. Technol..

[62] Santonicola S., Volgare M., Cocca M., Dorigato G., Giaccone V., Colavita G. (2023). Impact of Fibrous Microplastic
Pollution on Commercial
Seafood and Consumer Health: A Review. Animals.

[63] Fagiano V., Alomar C., Ventero A., de Puelles M. L. F., Iglesias M., Deudero S. (2024). First Assessment of
Anthropogenic
Particle Ingestion in Pontellid Copepods: Pontella Mediterranea as
a Potential Microplastic Reservoir in the Neuston. Sci. Total Environ..

[64] Middleton L., Wu W., Johnston T. M. S., Tarry D. R., Farrar J. T., Poulain P. M., Özgökmen T. M., Shcherbina A. Y., Pascual A., McNeill C. L. (2025). Observations of a Splitting
Ocean Cyclone Resulting in Subduction of Surface Waters. Sci. Adv..

[65] Martin
Bland J., Altman D. G. (1986). Statistical Methods for Assessing
Agreement between Two Methods of Clinical Measurement. Lancet.

[66] Zhu X., Nguyen B., You J. B., Karakolis E., Sinton D., Rochman C. (2019). Identification of Microfibers
in
the Environment Using Multiple Lines of Evidence. Environ. Sci. Technol..

[67] Prata J. C., Castro J. L., da Costa J. P., Duarte A. C., Cerqueira M., Rocha-Santos T. (2020). An Easy Method
for Processing and Identification of
Natural and Synthetic Microfibers and Microplastics in Indoor and
Outdoor Air. MethodsX.

[68] Primpke S., Dias P. A., Gerdts G. (2019). Automated Identification
and Quantification
of Microfibres and Microplastics. Anal. Methods.

[69] Kruskal W. H., Wallis W. A. (1952). Use of Ranks in
One-Criterion Variance Analysis. J. Am. Stat.
Assoc.

[70] Parsons, T. R. ; Maita, Y. ; Lalli, C. M. A Manual of Chemical and Biological Methods for Seawater Analysis; Pergamon Press: Oxford, UK, 1984.

[71] Sun Q., Little C. M., Barthel A. M., Padman L. (2021). A Clustering-Based
Approach to Ocean Model-Data Comparison around Antarctica. Ocean Sci..

[72] Virmani, D. ; Taneja, S. ; Malhotra, G. Normalization Based K Means Clustering Algorithm. arXiv 2015. 10.48550/arXiv.1503.00900.

[73] Lilliefors H. W. (1967). On the
Kolmogorov-Smirnov Test for Normality with Mean and Variance Unknown. J. Am. Stat. Assoc..

[74] Mann H. B., Whitney D. R. (1947). On a Test of Whether
One of Two Random Variables Is
Stochastically Larger than the Other. Ann. Math.
Stat..

[75] Juza M., Renault L., Ruiz S., Tintoré J. (2013). Origin and
Pathways of Winter Intermediate Water in the Northwestern Mediterranean
Sea Using Observations and Numerical Simulation. J. Geophys. Res. Ocean.

[76] Soto-Navarro J., Jordá G., Deudero S., Alomar C., Amores Á., Compa M. (2020). 3D Hotspots
of Marine Litter in the Mediterranean:
A Modeling Study. Mar. Pollut. Bull..

[77] De
La Fuente R., Drótos G., Hernández-García E., López C., Van Sebille E. (2021). Sinking Microplastics in the Water
Column: Simulations in the Mediterranean Sea. Ocean Sci..

[78] Roblin B., Ryan M., Vreugdenhil A., Aherne J. (2020). Ambient Atmospheric
Deposition of Anthropogenic Microfibers and Microplastics on the Western
Periphery of Europe (Ireland). Environ. Sci.
Technol..

[79] Kowalski N., Reichardt A. M., Waniek J. J. (2016). Sinking Rates of Microplastics and
Potential Implications of Their Alteration by Physical, Biological,
and Chemical Factors. Mar. Pollut. Bull..

[80] Francalanci S., Paris E., Solari L. (2021). On the Prediction of Settling Velocity
for Plastic Particles of Different Shapes. Environ.
Pollut..

[81] Sutherland B. R., Dibenedetto M., Kaminski A., Van Den Bremer T. (2023). Fluid Dynamics
Challenges in Predicting Plastic Pollution Transport in the Ocean:
A Perspective. Phys. Rev. Fluids.

[82] Maxey M. R., Riley J. J. (1983). Equation of Motion
for a Small Rigid Sphere in a Nonuniform
Flow. Phys. Fluids.

[83] Monroy P., Hernández-García E., Rossi V., López C. (2017). Modeling the
Dynamical Sinking of Biogenic Particles in Oceanic Flow. Nonlinear Process. Geophys..

[84] Qi G., Nathan G. J., Kelso R. M. (2014). The Influence
of Aspect Ratio on
Distributions of Settling Velocities and Orientations of Long Fibres. Powder Technol..

[85] Harcourt R. R., D’Asaro E. A. (2008). Large-Eddy Simulation of Langmuir
Turbulence in Pure
Wind Seas. J. Phys. Oceanogr..

[86] D’Asaro E. A. (2008). Convection
and the Seeding of the North Atlantic Bloom. J. Mar. Syst..

[87] Tarry D. R., Ruiz S., Johnston T. M. S., Poulain P. M., Özgökmen T., Centurioni L. R., Berta M., Esposito G., Farrar J. T., Mahadevan A. (2022). Drifter Observations Reveal Intense Vertical
Velocity in a Surface Ocean Front. Geophys.
Res. Lett..

[88] Esposito G., Donnet S., Berta M., Shcherbina A. Y., Freilich M., Centurioni L., D’Asaro E. A., Farrar J. T., Johnston T. M. S., Mahadevan A. (2023). Inertial Oscillations and Frontal Processes in an Alboran Sea Jet:
Effects on Divergence and Vertical Transport. J. Geophys. Res. Ocean.

[89] Donnet S., Huntley H. S., Berta M., Centurioni L., Middleton L., Özgökmen T., Poulain P.-M., Kinsella A., Griffa A. (2025). Surface evolution and wind effects
during a cyclonic eddy splitting event in the Balearic Sea. Ocean Sci..

[90] Pilo G. S., Oke P. R., Coleman R., Rykova T., Ridgway K. (2018). Patterns of
Vertical Velocity Induced by Eddy Distortion in an Ocean Model. J. Geophys. Res. Ocean.

[91] Gula J., Molemaker J. J., Mcwilliams J. C. (2014). Submesoscale Cold Filaments in the
Gulf Stream. J. Phys. Oceanogr..

[92] Dauhajre D. P., McWilliams J. C. (2018). Diurnal Evolution of Submesoscale Front and Filament
Circulations. J. Phys. Oceanogr..

[93] Rypina I. I., Pratt L. J., Dotzel M. (2024). Aggregation of Slightly Buoyant Microplastics
in 3D Vortex Flows. Nonlinear Process. Geophys..

[94] Juza M., Mourre B., Renault L., Gómara S., Sebastián K., Lora S., Beltran J. P., Frontera B., Garau B., Troupin C., Torner M., Heslop E., Casas B., Escudier R., Vizoso G., Tintoré J. (2016). SOCIB Operational
Ocean Forecasting System and Multi-Platform Validation in the Western
Mediterranean Sea. J. Oper. Oceanogr..

[95] Zhang J., Choi C. E. (2022). Improved Settling
Velocity for Microplastic Fibers:
A New Shape-Dependent Drag Model. Environ. Sci.
Technol..

[96] Marchetti B., Raspa V., Lindner A., Du Roure O., Bergougnoux L., Guazzelli É., Duprat C. (2018). Deformation of a Flexible Fiber Settling
in a Quiescent Viscous Fluid. Phys. Rev. Fluids.

[97] Coll M., Bellido J. M., Pennino M. G., Albo-Puigserver M., Báez J. C., Christensen V., Corrales X., Fernández-Corredor E., Giménez J., Julià L., Lloret-Lloret E., Macias D., Ouled-Cheikh J., Ramírez F., Sbragaglia V., Steenbeek J. (2024). Retrospective
Analysis of the Pelagic
Ecosystem of the Western Mediterranean Sea: Drivers, Changes and Effects. Sci. Total Environ..

[98] Fanelli E., Da Ros Z., Menicucci S., Malavolti S., Biagiotti I., Canduci G., De Felice A., Leonori I. (2023). The Pelagic Food Web of the Western Adriatic Sea: A
Focus on the Role of Small Pelagics. Sci. Rep..

[99] Compa M., Alomar C., Ventero A., Iglesias M., Deudero S. (2022). Anthropogenic
Particles in the Zooplankton Aggregation Layer and Ingestion in Fish
Species along the Catalan Continental Shelf. Estuar. Coast. Shelf Sci..

[100] Santini S., De Beni E., Martellini T., Sarti C., Randazzo D., Ciraolo R., Scopetani C., Cincinelli A. (2022). Occurrence
of Natural and Synthetic Micro-Fibers in
the Mediterranean Sea: A Review. Toxics.

[101] Concato M., Panti C., Baini M., Galli M., Giani D., Fossi M. C. (2023). Detection of Anthropogenic
Fibres
in Marine Organisms: Knowledge Gaps and Methodological Issues. Mar. Pollut. Bull..

[102] Guzzetti E., Sureda A., Tejada S., Faggio C. (2018). Microplastic
in Marine Organism: Environmental and Toxicological Effects. Environ. Toxicol. Pharmacol..

[103] Botterell Z. L. R., Beaumont N., Dorrington T., Steinke M., Thompson R. C., Lindeque P. K. (2019). Bioavailability
and Effects of Microplastics on Marine Zooplankton: A Review. Environ. Pollut..

[104] Coppock R. L., Galloway T. S., Cole M., Fileman E. S., Queirós A. M., Lindeque P. K. (2019). Microplastics Alter Feeding Selectivity
and Faecal Density in the Copepod, Calanus Helgolandicus. Sci. Total Environ..

[105] Zhao M., Huang L., Babu Arulmani S. R., Yan J., Wu L., Wu T., Zhang H., Xiao T. (2022). Adsorption
of Different Pollutants by Using Microplastic with Different Influencing
Factors and Mechanisms in Wastewater: A Review. Nanomaterials.

[106] Gove J. M., Whitney J. L., McManus M. A., Lecky J., Carvalho F. C., Lynch J. M., Li J., Neubauer P., Smith K. A., Phipps J. E., Kobayashi D. R., Balagso K. B., Contreras E. A., Manuel M. E., Merrifield M. A., Polovina J. J., Asner G. P., Maynard J. A., Williams G. J. (2019). Prey-Size
Plastics Are Invading Larval Fish Nurseries. Proc. Natl. Acad. Sci. U. S. A..

