# Supplementary material for: Microfibers Accumulation within a Mediterranean Submesoscale Cyclone
Source: Environ Sci Technol. 2026 Jan 14;60(3):2639–48. doi: 10.1021/acs.est.5c13987 (PMC12854745; doi:10.1021/acs.est.5c13987)
Supplement: Supplementary file 1 [file es5c13987_si_001.pdf]

Supplementary Information

Microfibers accumulation within a Mediterranean submesoscale cyclone

Giovanni Testa[§]*, Giuseppe Suaria[§], Andrea Paluselli, Salomé La Ragione, Michela Gambale, Maristella Berta, Lorena A. Rivera, Amala Mahadevan, Leo Middleton, Francesco M. Falcieri, Stefano Aliani, Annalisa Griffa

[§] G.T. and G.S. contributed equally to this work
*Corresponding author

Contents:

- 11 pages
- 7 figures (Figures S1–S7)
- 2 tables (Tables S1–S2)

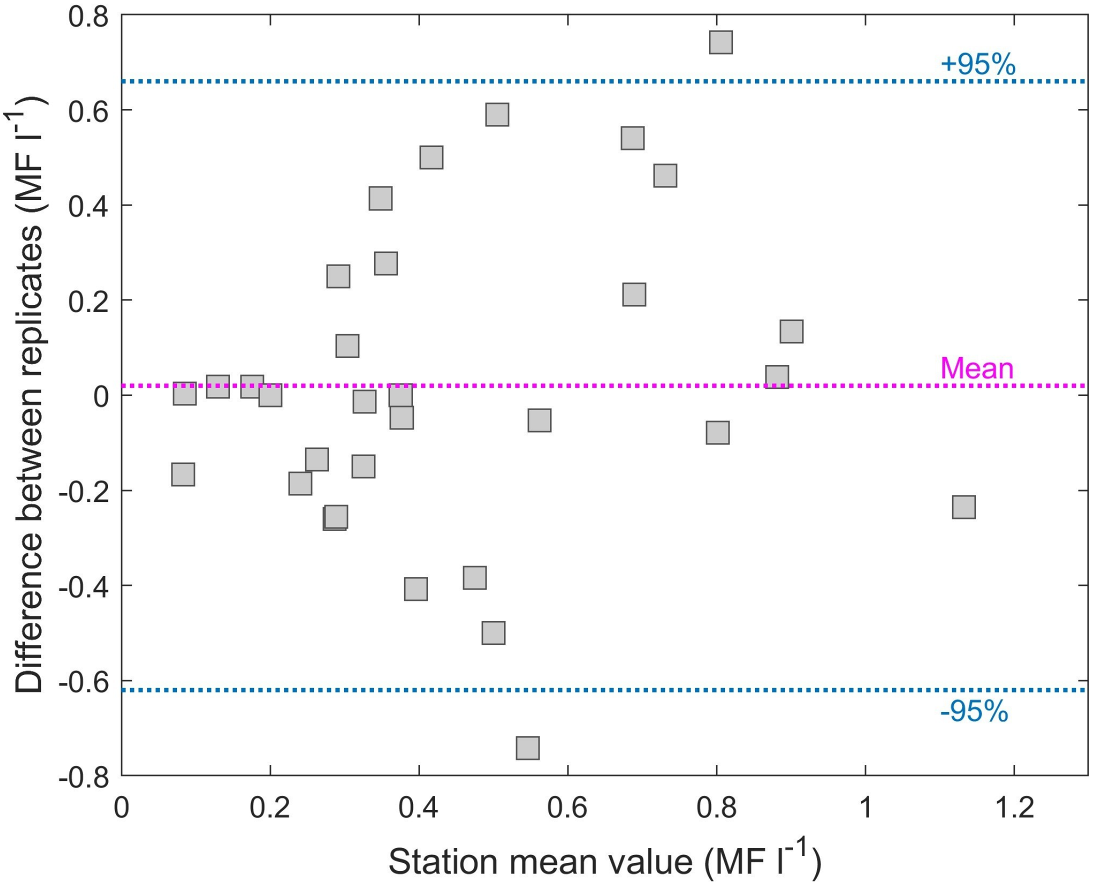

Figure S1. Consistency of microfiber replicates. Bland-Altman plot showing the mean microfiber concentration values obtained from the two replicates collected at each depth and the corresponding difference between replicates. The magenta dotted line indicates the mean difference, while the blue dotted lines represent the 95% limits of agreement for the differences between replicates.

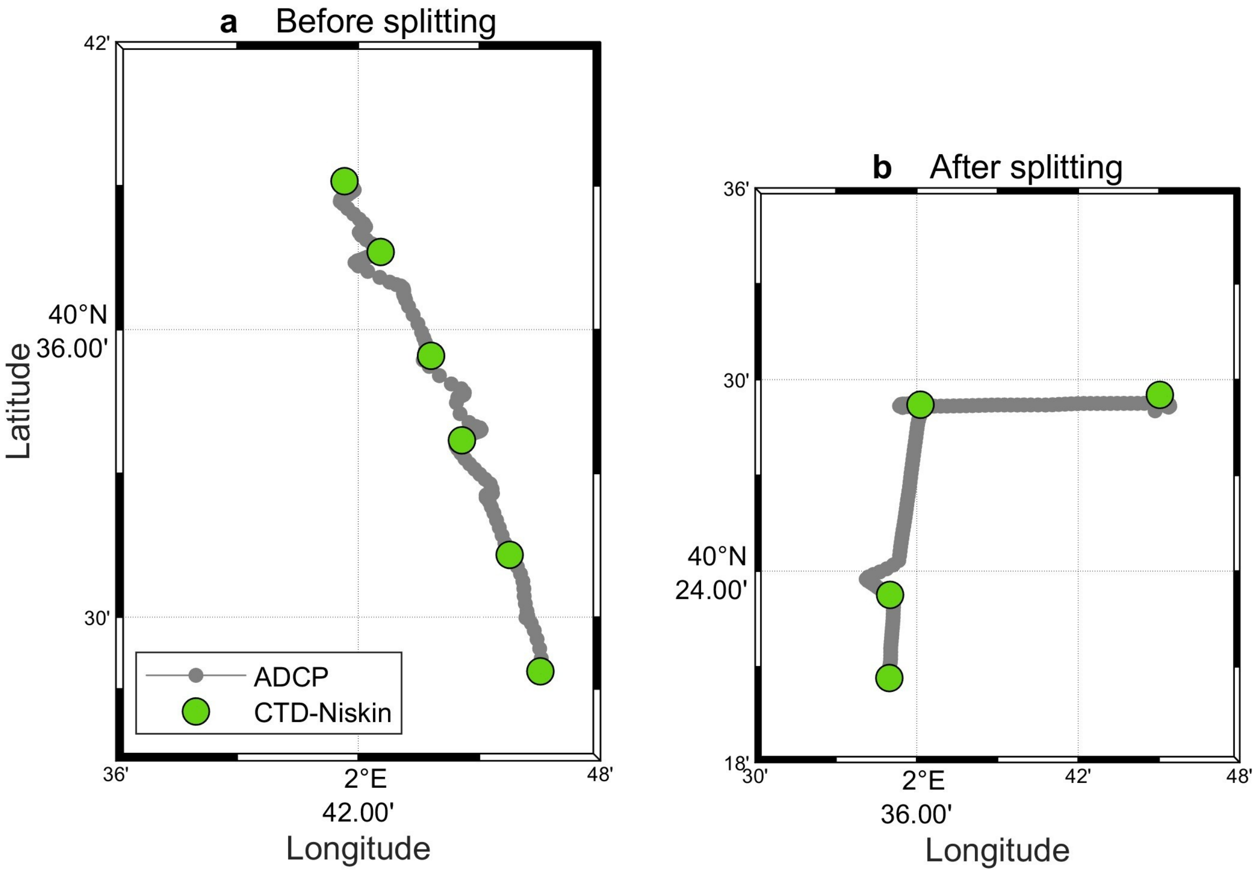

Figure S2. Sampling locations of ADCP and CTD stations. a Geographical location of ADCP (gray) and CTD-Niskin (green) stations sampled along the transect conducted before the eddy split during the 2022 CALYPSO campaign. **b** Same as panel a, but for the transect realized after the eddy split.

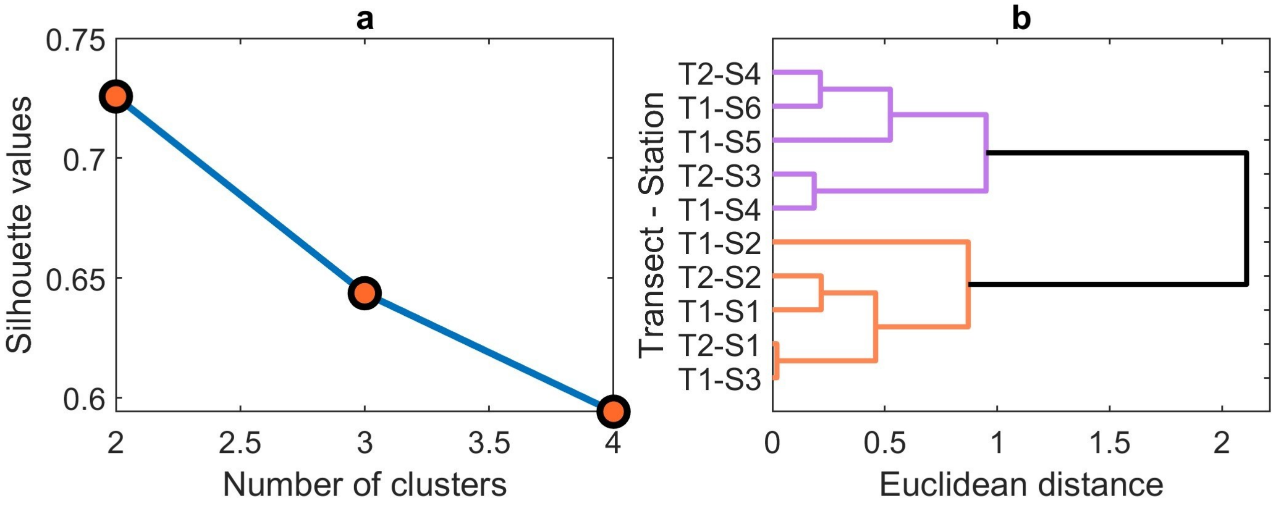

Figure S3. Clustering of oceanographic stations. a Silhouette analysis. **b** Dendrogram illustrating the division of the 10 oceanographic stations. T1: transect in the eddy before the split; T2: transect in eddy after the split. Node colors in the dendrogram indicate station location: orange for stations inside the eddy, purple for stations outside the eddy.

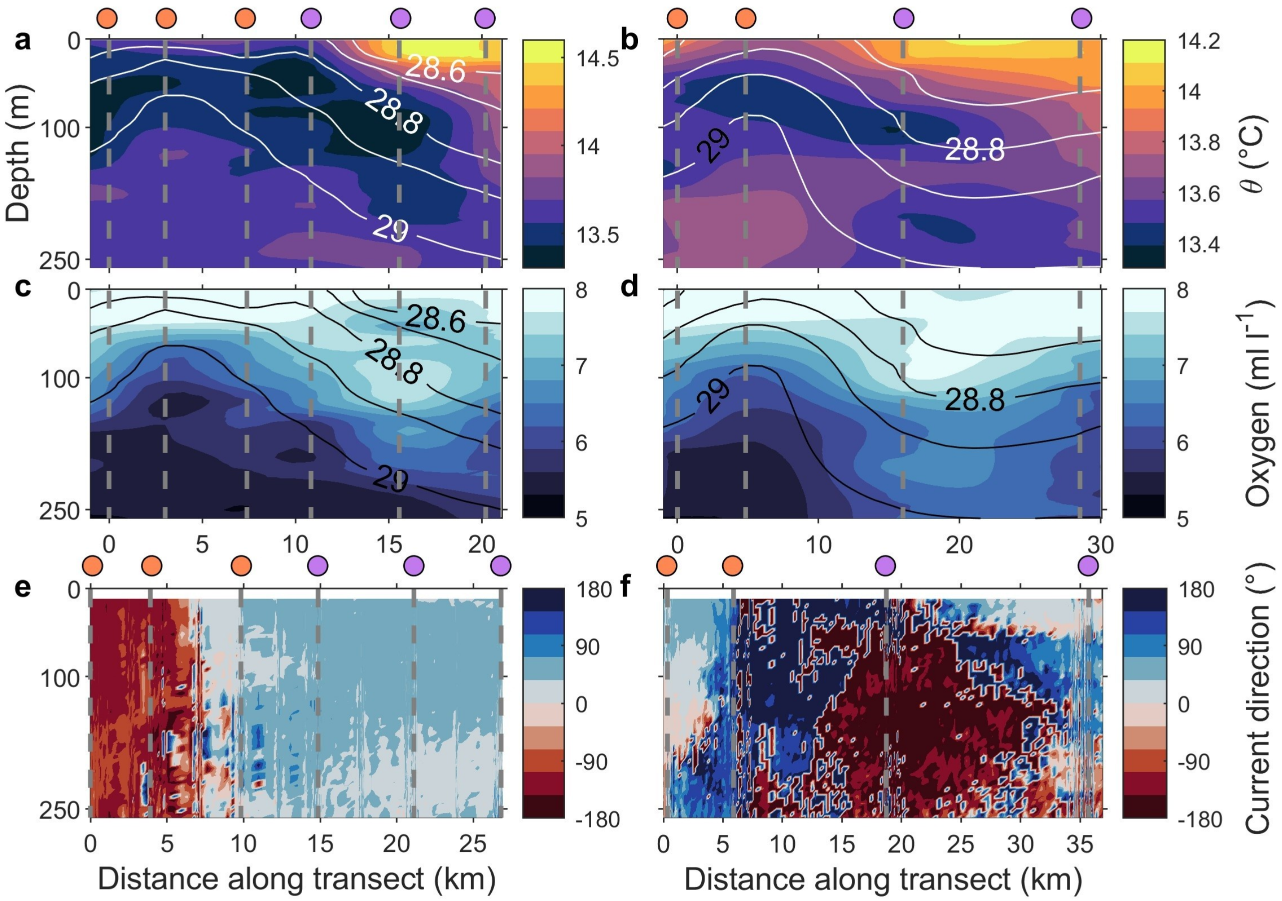

Figure S4. Profiles of temperature, dissolved oxygen, and current direction along the two transects. a-b Conservative Temperature. **c-d** Dissolved oxygen. **e-f** Current direction. The left (right) panels show the transects in the eddy before (after) the split. Contours represent potential density anomaly. Station location marked by gray dashed lines, with colored markers above the upper panels indicate whether the stations were located inside (orange) or outside (purple) the cyclone. Conservative Temperature and dissolved oxygen were measured from a Conductivity Temperature Depth (CTD) profiler, while current direction was measured with an Acoustic Doppler Currents Profiler (ADCP). Note that the distances along the transects differ between the CTD/Niskin data (a-d) and ADCP data (e-f) due to variations in the ship's course and sampling frequencies (Figure S2).

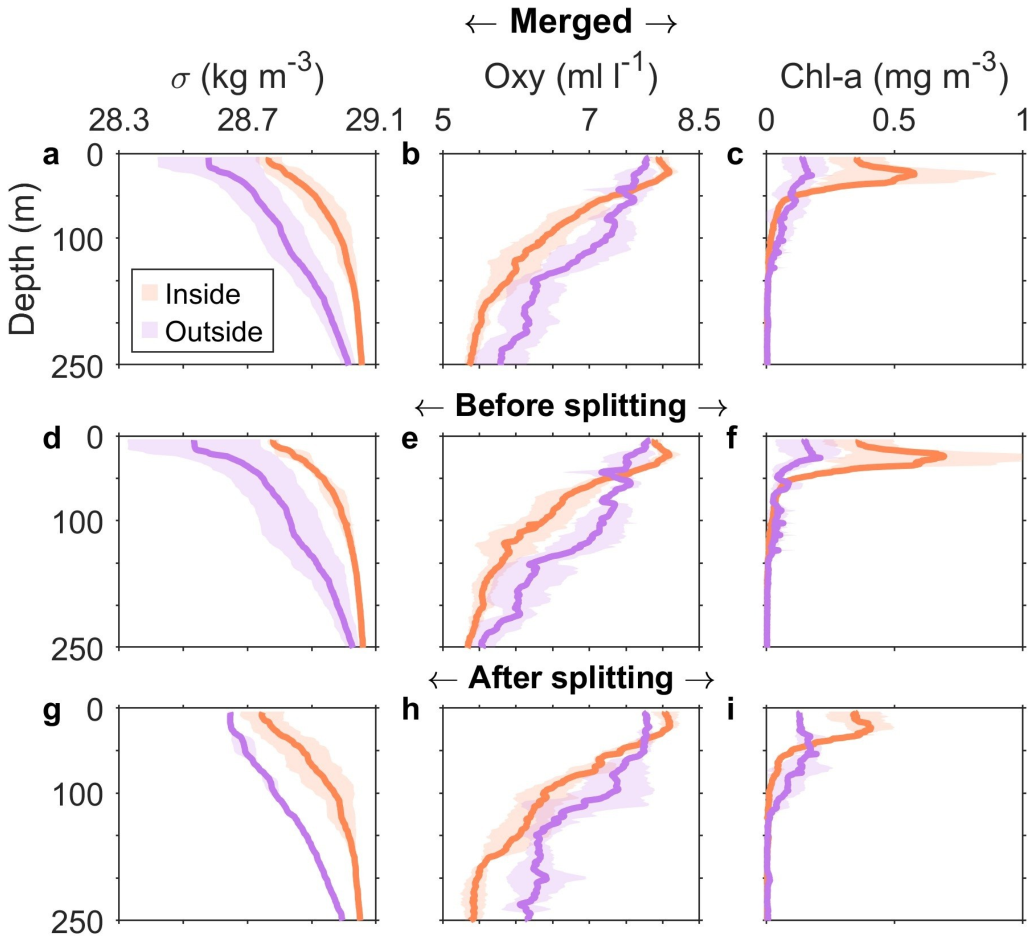

Figure S5. Density anomaly, dissolved oxygen, and chlorophyll-a concentrations inside and outside the cyclones. **a-c** Mean values (solid line) and 95% confidence interval (shaded area) for the combined data from both transects. **d-f** Same as panels a-c, but showing data from the transect conducted before the eddy split. **g-i** Same as panels a-c, but showing data from the transect conducted after the eddy split. Orange and purple colors represent data collected inside and outside the cyclones, respectively.

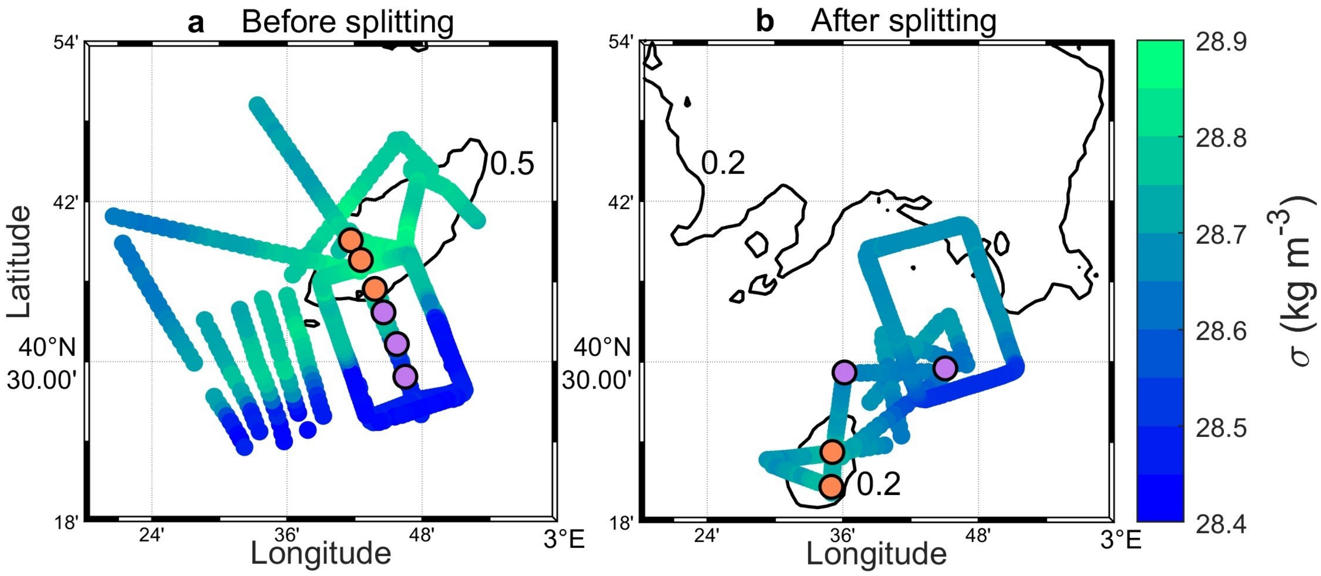

Figure S6. Density front observed in the study zone. a Potential density anomaly values at 10 m depth collected between 22nd and 24th February with an Underway CTD probe. **b** Same as panel a, but for data obtained between 28th of February and 1st of March. Contours represent the chlorophyll-a isolines equal to 0.5 and 0.2 mg m^{-3}, whereas the orange and purple points represent MF sampling stations classified as being located inside and outside the cyclone, respectively.

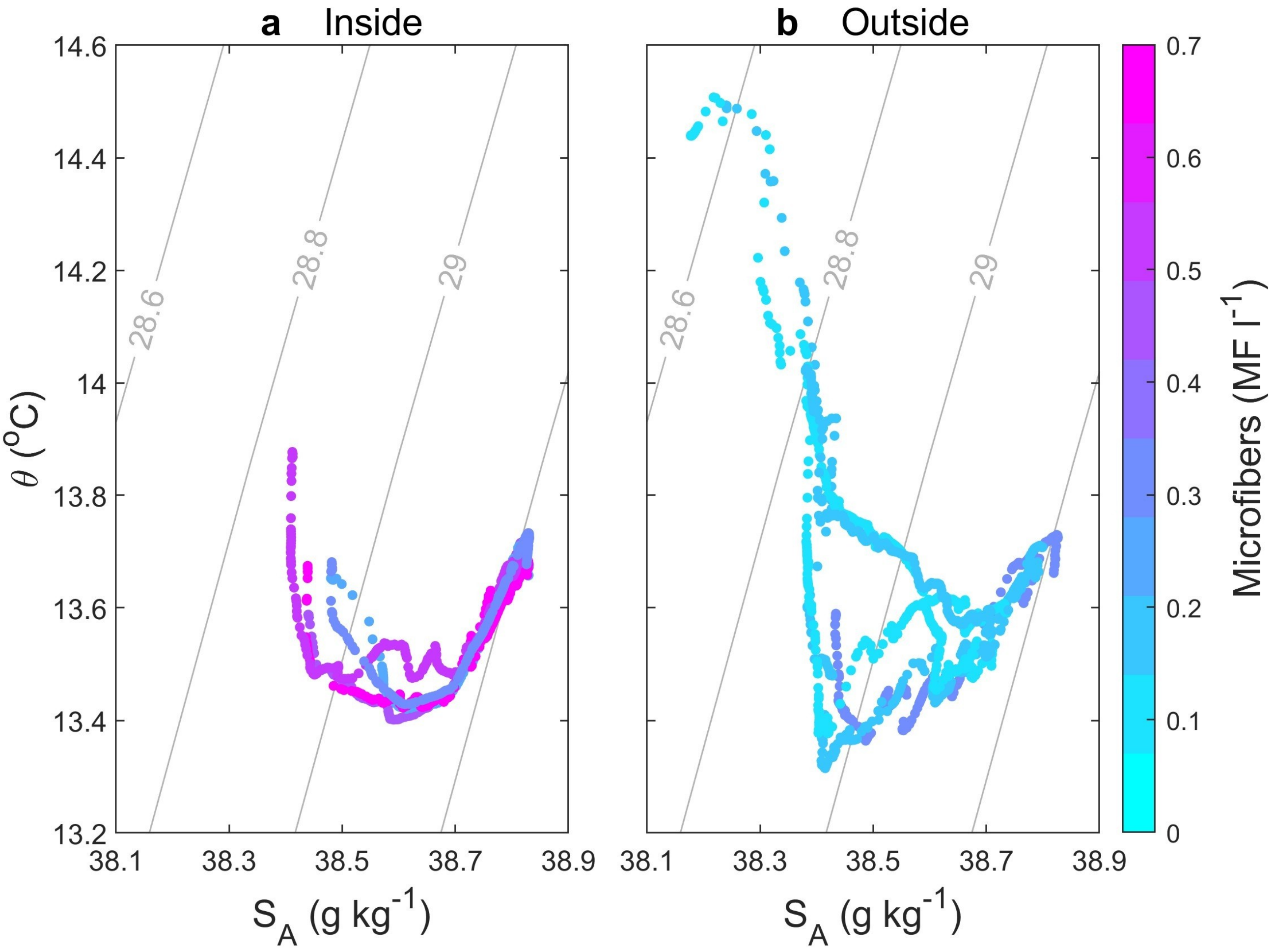

Figure S7. Temperature-salinity diagrams for the stations located inside and outside the cyclone. a Potential temperature- Absolute Salinity diagram for the stations located 'inside' the cyclone. **b** Same as panel a, but for stations located 'outside' the cyclone. Each profile was colored according to its mean MF concentration.

Table S1. Microfiber concentrations before and after blank correction. Stations are identified by transect (B: sampled before eddy splitting; A: sampled after splitting) and position (IN: inside cyclone; OUT: outside cyclone). Depth indicates the sampling depth in meters.

Station	Transect	Depth	Microfibers (MF L^{-1})	
(n)		(m)	Uncorrected	Blank-corrected
12	B-IN	5	0.38	0.21
12	B-IN	25	0.90	0.74
12	B-IN	75	0.26	0.10
12	B-IN	150	0.69	0.52
12	B-IN	205	0.80	0.64
13	B-IN	5	0.20	0.03
13	B-IN	15	0.33	0.16
13	B-IN	25	0.51	0.34
13	B-IN	53	0.18	0.01
13	B-IN	150	0.69	0.52
13	B-IN	204	0.47	0.31
14	B-IN	5	0.40	0.31
14	B-IN	25	0.81	0.72
14	B-IN	35	1.13	1.05
14	B-IN	123	0.73	0.65
14	B-IN	204	0.88	0.80
15	B-OUT	5	0.13	0.13
15	B-OUT	17	0.38	0.38
15	B-OUT	53	0.09	0.09
15	B-OUT	75	0.29	0.29
15	B-OUT	150	0.29	0.29
15	B-OUT	206	0.56	0.56
16	B-OUT	5	0.50	0.25
16	B-OUT	18	0.36	0.10
16	B-OUT	44	0.30	0.05
16	B-OUT	60	0.42	0.16
16	B-OUT	105	0.49	0.24
16	B-OUT	201	0.55	0.29
17	B-OUT	5	0.20	0.03
17	B-OUT	25	0.35	0.18
17	B-OUT	48	0.29	0.12
17	B-OUT	77	0.33	0.16
17	B-OUT	179	0.08	0.00
17	B-OUT	228	0.24	0.07
31	A-IN	5	0.20	0.12
31	A-IN	27	0.40	0.32
31	A-IN	70	0.83	0.75
31	A-IN	105	2.00	1.92
31	A-IN	175	0.17	0.08
31	A-IN	200	0.17	0.08

-------------------------------- continue --------------------------------

32	A-IN	5	0.38	0.38
32	A-IN	25	0.30	0.30
32	A-IN	55	0.58	0.58
32	A-IN	105	0.42	0.42
32	A-IN	150	0.00	0.00
32	A-IN	200	0.33	0.33
33	A-OUT	5	0.00	0.00
33	A-OUT	33	0.14	0.00
33	A-OUT	50	0.70	0.45
33	A-OUT	72	0.25	0.00
33	A-OUT	113	0.25	0.00
33	A-OUT	200	0.00	0.00
34	A-OUT	5	0.17	0.08
34	A-OUT	50	0.11	0.03
34	A-OUT	90	0.00	0.00
34	A-OUT	160	0.17	0.08
34	A-OUT	200	0.17	0.08
34	A-OUT	250	0.67	0.58

Table S2. Microfibers composition measured by µFTIR. Microfibers classification as material, sub-material, the percentage relative to the total microfiber dataset and their relative densities are shown (n = 171).

Material	Sub-material	Percentage (%)	Density (g cm^{-3})
Cellulosic		91.7	1.4-1.56
	Cotton	55.9	
	Rayon/Viscose	3.4	
	Other cellulosic fibers	32.4	
Synthetic	Polyester, acrylic, polypropylene	4.8	0.9-1.3
Animal	Wool	3.4	1.3
